# Evidence Based Selection of Housekeeping Genes

**DOI:** 10.1371/journal.pone.0000898

**Published:** 2007-09-19

**Authors:** Hendrik J. M. de Jonge, Rudolf S. N. Fehrmann, Eveline S. J. M. de Bont, Robert M. W. Hofstra, Frans Gerbens, Willem A. Kamps, Elisabeth G. E. de Vries, Ate G. J. van der Zee, Gerard J. te Meerman, Arja ter Elst

**Affiliations:** 1 Division of Pediatric Oncology/Hematology, Department of Pediatrics, Beatrix Children's Hospital, University Medical Center Groningen, University of Groningen, Groningen, The Netherlands; 2 Department of Genetics, University Medical Center Groningen, University of Groningen, Groningen, The Netherlands; 3 Department of Gynecology, University Medical Center Groningen, University of Groningen, Groningen, The Netherlands; 4 Department of Medical Oncology, University Medical Center Groningen, University of Groningen, Groningen, The Netherlands; National Cancer Institute, United States of America

## Abstract

For accurate and reliable gene expression analysis, normalization of gene expression data against housekeeping genes (reference or internal control genes) is required. It is known that commonly used housekeeping genes (e.g. *ACTB*, *GAPDH*, *HPRT1*, and *B2M*) vary considerably under different experimental conditions and therefore their use for normalization is limited. We performed a meta-analysis of 13,629 *human* gene array samples in order to identify the most stable expressed genes. Here we show novel candidate housekeeping genes (e.g. *RPS13*, *RPL27, RPS20* and *OAZ1*) with enhanced stability among a multitude of different cell types and varying experimental conditions. None of the commonly used housekeeping genes were present in the top 50 of the most stable expressed genes. In addition, using 2,543 diverse *mouse* gene array samples we were able to confirm the enhanced stability of the candidate novel housekeeping genes in another mammalian species. Therefore, the identified novel candidate housekeeping genes seem to be the most appropriate choice for normalizing gene expression data.

## Introduction

Measuring transcript abundance by real-time reverse transcription PCR (RT-PCR) has become the method of choice due to its high sensitivity, specificity and broad quantification range for high-throughput and accurate expression profiling of selected genes.[1] RT-PCR is the most commonly used method for molecular diagnostics, validating microarray data of a smaller set of genes and is especially useful when only a small number of cells is available.[2]–[6] Besides being a powerful technique RT-PCR suffers from certain pitfalls, with inappropriate data normalization as the most important problem. Various strategies have been applied to control gene expression results. Standardization of the amount of cells is for instance a problem when tissue samples are used. Quantification of total RNA is difficult when only minimal RNA quantities are available. More importantly, it measures the total RNA fraction of a sample, which consists for only a relatively small percentage (<10%) of mRNA and predominantly of rRNA molecules. A drawback to the use of 18S or 28S rRNA molecules as control genes is the abovementioned imbalance between mRNA and rRNA fractions.[7] In addition, it has been shown that certain biological factors and drugs may affect rRNA transcription.[8], [9] Finally, those approaches still do not take a correction for the efficiency of enzymatic reactions into account. At this moment housekeeping genes are the gold standard to normalize the mRNA fraction. However, the known considerable variation in gene expression of commonly used housekeeping genes will add noise to an experiment and could ultimately lead to erroneous results.[10]–[12] This even resulted in strategies to control for the instability by using sets of control genes and calculation of normalization factors using statistical algorithms.[1], [12], [13] In order to identify the most stable expressed housekeeping genes we used a large set of expression data from 13,629 published human gene arrays and investigated the abundance and stability in gene expression levels. We validated the human results in mice using a set of 2,543 published mouse gene arrays.

## Results and Discussion

**Figure 1:**
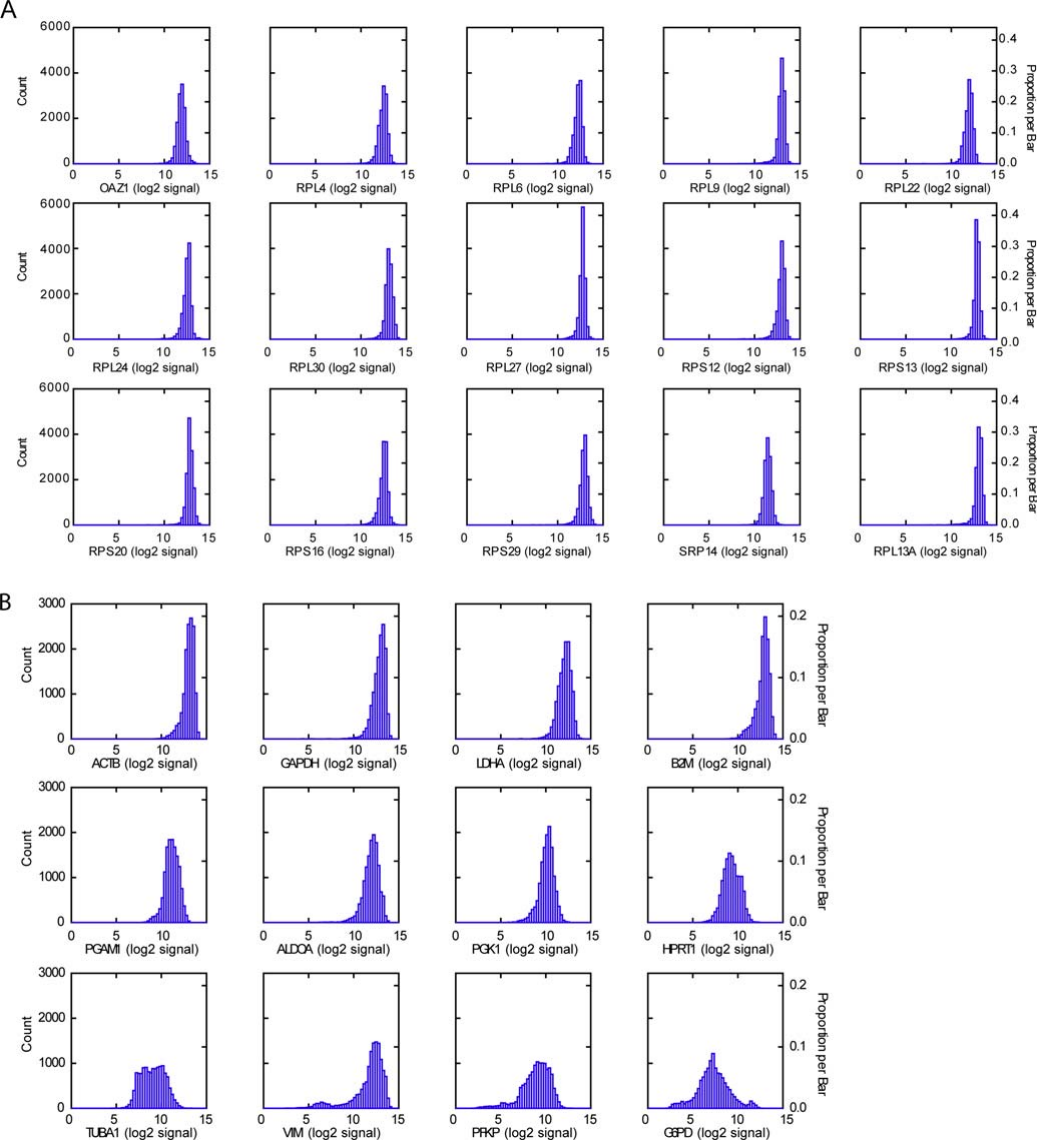
Expression distribution of the top 15 candidate housekeeping genes (A) and of 12 commonly used housekeeping genes in 13,629 human samples (B).

A candidate housekeeping gene was defined as a gene with the most stable expression, i.e. a gene with a small coefficient of variation (CV) and a maximum fold change <2 (MFC, the ratio of the maximum and minimum values observed within the dataset). In addition, a mean expression level lower than the maximum expression level subtracted with 2 standard deviation (SD) was a prerequisite for a candidate housekeeping gene. The expression levels of 13,037 unique genes in the set of 13,629 diverse samples were used. Table 1 shows the identified top 15 candidate housekeeping genes (Table S1 shows CVs of all 13,037 unique genes). All 15 genes had a CV beneath the 4% level and a standard deviation below 0.49. Moreover, the MFCs ranged from 1.41 (*RPL27*) to 1.99 (*RPS12*), reflecting the minor variation in expression of those candidate housekeeping genes within the large dataset. Thirteen of these top 15 genes encode for ribosomal proteins involved in protein biosynthesis. The distribution of the expression levels is given in Figure 1A.

**Table 1:** Top 15 candidate housekeeping genes identified in 13,629 samples.

Gene symbol	name	mean	SD	CV (%)	MFC	rank
*RPS13*	ribosomal protein S13	12.82	0.33	2.59	1.61	1
*RPL27*	ribosomal protein L27	12.70	0.35	2.73	1.41	2
*RPS20*	ribosomal protein S20	12.81	0.37	2.90	1.67	3
*RPL30*	ribosomal protein L30	13.08	0.42	3.22	1.99	4
*RPL13A*	ribosomal protein L13A	13.01	0.43	3.29	1.83	5
*RPL9*	ribosomal protein L9	12.95	0.44	3.36	1.68	6
*SRP14*	signal recognition particle 14kDa	11.45	0.40	3.46	1.48	7
*RPL24*	ribosomal protein L24	12.50	0.46	3.65	1.54	8
*RPL22*	ribosomal protein L22	11.94	0.44	3.68	1.91	9
*RPS29*	ribosomal protein S29	12.86	0.47	3.69	1.93	10
*RPS16*	ribosomal protein S16	12.48	0.47	3.73	1.62	11
*RPL4*	ribosomal protein L4	12.43	0.47	3.76	1.63	12
*RPL6*	ribosomal protein L6	12.22	0.46	3.76	1.65	13
*OAZ1*	ornithine decarboxylase antizyme 1	11.88	0.45	3.78	1.51	14
*RPS12*	ribosomal protein S12	12.90	0.49	3.82	1.99	15

CV, indicates the coefficient of variation and equals the standard deviation divided by the mean (expressed as a percentage). MFC, indicates the maximum fold change, i.e. the ratio of the maximum and minimum values observed within a dataset. The ranking is based upon three criteria: CV, a MFC<2 and a mean value lower then the maximum value with 2 standard deviation (SD) subtracted.

Next, we studied the expression levels of commonly used housekeeping genes (e.g. *ACTB*, *GAPDH*, *HPRT1* and *B2M*). The expression levels of those commonly used housekeeping genes fluctuated dramatically (Table 2). The MFC ranged from 1.91 (*ACTB*) to 15.15 (*ALDOA*). Moreover, for only one of 12 commonly used housekeeping genes (*ACTB*) the CV was beneath the 5% level, reflecting the highly variable levels of those commonly used housekeeping genes within our large dataset. Remarkably, none of the classical housekeeping genes ranked among the top 50 identified candidate housekeeping genes. The distribution of expression levels of commonly used housekeeping genes is depicted in Figure 1B.

**Table 2:** Ranking of 12 commonly used housekeeping genes identified in 13,629 samples.

Gene symbol	Name	mean	SD	CV (%)	MFC	rank
*ACTB*	β-actin	13.00	0.63	4.88	1.91	57
*GAPDH*	glyceraldehyde-3 phosphate dehydrogenase	12.83	0.74	5.75	6.37	139
*LDHA*	lactate dehydrogenase A	12.09	0.72	5.92	2.21	168
*B2M*	β-2-microglobulin	12.75	0.76	5.97	4.01	176
*PGAM1*	phosphoglycerate mutase	11.14	0.76	6.87	2.03	413
*ALDOA*	aldolase A	11.94	0.92	7.74	15.15	767
*PGK1*	phosphoglycerate kinase	10.08	0.82	8.17	2.19	996
*HPRT1*	hypoxanthine phosphoribosyl-transferase	9.29	0.92	9.94	2.48	2193
*TUBA1*	α-tubulin	9.04	1.28	14.15	2.87	4921
*VIM*	vimentin	11.65	1.87	16.01	5.83	6016
*PFKP*	phosphofructokinase	8.89	1.59	17.93	6.25	7019
*G6PD*	glucose-6 phosphate dehydrogenase	7.27	1.74	23.86	5.78	9707

CV, indicates the coefficient of variation and equals the standard deviation divided by the mean (expressed as a percentage). The ranking of these commonly used housekeeping genes among all 13,037 unique tested genes is based on the CV.

**Figure 2:**
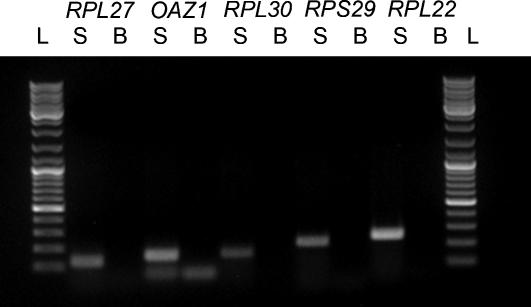
PCR results of 5 novel candidate housekeeping genes. S indicates sample, cDNA of a HL-60 leukemic cell line was used for all primers, B indicates the blanc (H_2_O) and L indicates the 100 base pair ladder (Fermentas).

To demonstrate the feasibility of the use of these novel candidate housekeeping genes, we created for 5 of the top 15 candidate housekeeping genes primers (i.e. *RPL27*, *RPL30*, *OAZ1*, *RPL22* and *RPS29*). We tested with PCR for desired product length and specificity; no pseudogenes were amplified (Figure 2 shows the PCR results).

To validate the enhanced stability of the identified novel candidate housekeeping genes we used another mammalian model system, i.e. the mouse. The expression levels of 21,377 unique genes in a set of 2,543 diverse mouse samples were used. The novel candidate housekeeping genes identified in the human data set also showed stability in expression in mouse arrays (Table 3). Also in mouse expression arrays genes encoding for ribosomal proteins are the most stable expressed ones. So, the stability in expression of the identified candidate housekeeping genes was confirmed in another species.

**Table 3:** The variation in expression of the candidate housekeeping genes in *mice*.

novel candidate housekeeping genes			
*gene symbol*	*SD*	*CV (%)*	*MFC*
*RPS29*	0.26	1.92	1.26
*RPL4*	0.39	2.95	1.34
*OAZ1*	0.43	3.42	1.34
*RPL13A*	0.50	3.89	1.36
*RPL6*	0.50	3.90	1.30
*SRP14*	0.56	5.22	1.40
*RPL24*	0.63	6.10	1.59
*RPL27*	0.74	6.16	1.53
*RPS13*	0.73	6.34	1.50
*RPL9*	0.57	6.41	1.56
*RPL22*	0.76	6.42	1.46
*RPS16*	0.80	6.46	1.49
*RPS12*	0.83	7.01	1.49
*RPS20*	1.01	8.61	1.57
*RPL30*	0.87	8.97	3.80

CV, indicates the coefficient of variation and equals the standard deviation divided by the mean (expressed as a percentage). MFC, indicates the maximum fold change, i.e. the ratio of the maximum and minimum values observed within a dataset.

Our results clearly reveal novel candidate housekeeping genes with a more stable expression in different cellular and experimental contexts in comparison to frequently used housekeeping genes (e.g. *ACTB*, *GAPDH* and *HPRT*). On the basis of a definition of ubiquitous and stable expression, our results indicate however that no single gene qualifies as a ‘real’ housekeeping gene. *GAPDH* and *ACTB* were used as single control genes in more then 90% of the cases in high impact journals.[11] Commonly used control genes are historical carryovers and were considered good references for many years in techniques where a qualitative change was being measured, because these genes are expressed at relatively high levels in nearly all cells. However, the advent of RT-PCR placed the emphasis on quantitative change, and asks for a re-evaluation of the use of these historical housekeeping genes. Here we show for the first time a genome wide evaluation of candidate housekeeping genes by a meta-analysis of more then 13,000 samples. Interestingly, the identified candidate novel housekeeping genes do not vary much in terms of functionality; they are predominantly ribosomal proteins involved in protein biosynthesis. Therefore, experimenters that tinker with this specific cellular process would better use other candidate housekeeping genes of our analysis, for example *OAZ1*.

Using meta-analysis we were able to find candidate housekeeping genes with a much lower level of variance in expression across tissue types and experimental conditions than commonly used housekeeping genes. Our identified candidate housekeeping genes can be applied in (nearly) all future RT-PCR experiments without any restrictions.

## Materials and Methods

Microarray expression data of 13,629 publicly available samples hybridized to Affymetrix HG-U133A and HG-U133 Plus 2.0 GeneChips (Affymetrix, Santa Clara, Ca.) were downloaded from the Gene Expression Omnibus.[14] This set of samples comprises gene expression data of a wide variety of different tissues (e.g. primary patient material, cell lines, diseased as well as normal tissues, stem cells etc.) and varying experimental conditions (e.g. transfected/transduced cells, cytokine stimulated, cells under hypoxic conditions, ultraviolet treated cells, cells treated with chemotherapeutics or non cytotoxic drugs etc.). Probesets that were available on both platforms were converted to official gene symbols, averaging expression values of multiple probesets targeting the same gene. Next, quantile normalization was applied to the log2 transformed expression values.[15] For each gene the CV of the expression was calculated. The CV equals the standard deviation divided by the mean (expressed as a percentage). The CV is used as a statistic for comparing the degree of variation between genes, even if the mean expressions are drastically different from each other.[16] The calculated CVs for all genes were ranked. In addition, the MFC was calculated to reflect the minor variation in expression of those candidate housekeeping genes within the large dataset. For validation 2,543 publicly available mouse samples hybridized to Affymetrix Mouse Genome 430 2.0 GeneChips (Affymetrix) were downloaded from the Gene Expression Omnibus.[14]. Again, this validation set comprises a wide variety of different mouse tissues and varying experimental conditions.

Total RNA was extracted with Absolutely RNA Miniprep Kit (Stratagene, Amsterdam, The Netherlands), and reverse-transcribed to cDNA with random hexamer and RevertAid^TM^ M-MuLV Reverse Transcriptase (Fermentas, Burlington, Ontario, Canada) according to the manufacturer's protocols. Table 4 shows primer sequences for *RPL27*, *RPL30*, *OAZ1*, *RPL22* and *RPS29.* The same annealing temperature (i.e. 60 °C) and number of cycles (i.e. 25) was used for all primers. The PCR products were analyzed by electrophoresis in a 1.0% agarose gel.

**Table 4:** Primer sequences of 5 candidate housekeeping genes.

Gene symbol	Forward	Reverse	Base pairs	T
*RPL27*	ATCGCCAAGAGATCAAAGATAA	TCTGAAGACATCCTTATTGACG	123	60
*RPL30*	ACAGCATGCGGAAAATACTAC	AAAGGAAAATTTTGCAGGTTT	158	60
*OAZ1*	GGATCCTCAATAGCCACTGC	TACAGCAGTGGAGGGAGACC	150	60
*RPL22*	TCGCTCACCTCCCTTTCTAA	TCACGGTGATCTTGCTCTTG	250	60
*RPS29*	GCACTGCTGAGAGCAAGATG	ATAGGCAGTGCCAAGGAAGA	213	60

Forward and reverse indicate the specific primers; base pairs, the product length and T, the annealing temperature given as °C.

## Supporting Information

Table S1 The CVs of all 13,037 unique genes in 13,629 samples.
(0.72 MB DOC)
